# Novel 3D organotypic co-culture model of pleura

**DOI:** 10.1371/journal.pone.0276978

**Published:** 2022-12-01

**Authors:** Isabella B. Metelmann, Sebastian Kraemer, Matthias Steinert, Stefan Langer, Peggy Stock, Olga Kurow

**Affiliations:** 1 Department of Visceral, Transplant, Thoracic and Vascular Surgery, University Hospital of Leipzig, Leipzig, Germany; 2 Department of Orthopedics, Trauma and Plastic Surgery, University Hospital Leipzig, Leipzig, Germany; National Cheng Kung University, TAIWAN

## Abstract

Pleural mesothelial cells are the predominant cell type in the pleural cavity, but their role in the pathogenesis of pleural diseases needs to be further elucidated. 3D organotypic models are an encouraging approach for an *in vivo* understanding of molecular disease development. The aim of the present study was to develop a 3D organotypic model of the pleural mesothelium. Specimens of human *pleura parietalis* were obtained from patients undergoing surgery at the University Hospital Leipzig, Germany. 3D co-culture model of pleura was established from human pleural mesothelial cells and fibroblasts. The model was compared to human pleura tissue by phase-contrast and light microscopy, immunochemistry and -fluorescence as well as solute permeation test. Histological assessment of the 3D co-culture model displayed the presence of both cell types mimicking the morphology of the human pleura. Vimentin and Cytokeratin, PHD1 showed a similar expression pattern in pleural biopsies and 3D model. Expression of Ki-67 indicates the presence of proliferating cells. Tight junctional marker ZO-1 was found localized at contact zones between mesothelial cells. Each of these markers were expressed in both the 3D co-culture model and human biopsies. Permeability of 3D organotypic co-culture model of pleura was found to be higher for 70 kDa-Dextran and no significant difference was seen in the permeability for small dextran (4 kDa). In summary, the presented 3D organoid of pleura functions as a robust assay for pleural research serving as a precise reproduction of the *in vivo* morphology and microenvironment.

## Introduction

Infectious and malignant pleural diseases, such as pleural empyema, carcinosis or mesothelioma, are severe illnesses that are associated with a high clinical impact. Pleural empyema, most commonly caused by pneumonia, cancer or iatrogenic disorder, is an increasing condition [1–3] often with a need for surgical interventions [4]. Relevant incidence of long-term consequences poses a significant burden on health sector. While substantial work has previously been done in the understanding of COVID-pneumonia, little is known about the pleural damage [5, 6]. End-stage disease of various carcinoma types, especially adenocarcinoma of the lung, mamma, or ovaries, can lead to a diffuse metastatic spread of the pleura, so called pleura carcinosis. Extended pleural effusion can lead to acute dyspnea and pain or even respiratory insufficiency and has a significant impact on patients’ wellbeing. Median survival of patients with pleural carcinosis is 12 months [7–9]. While all these diseases prove the significant healthcare burden of pleural illnesses, its scientific consideration is quite low. An in-depth understanding of pathogenesis is important for therapeutic treatment and prevention of these conditions.

Pleural mesothelial cells (PMCs) are the predominant cell type in the pleural cavity, but their role in the pathogenesis of pleural diseases needs to be further elucidated. In recent years, data from several groups have suggested the regenerative potential of PMCs and they role as progenitor cells after pleural or lung damage [10, 11]. PMCs display various immune functions and represent the central component of pleural defense mechanisms as a protective non-adhesive surface [12, 13]. They are also involved in transport of solutes and cells across serosal cavities, during inflammation and tumor cell adhesion [14]. Therefore, PMCs play a critical role in pleural and systemic diseases such as empyema, granulomatous diseases of the pleura and malignant pleural diseases [15].

For better understanding of the various functions of PMCs in order to develop effective therapies for these diseases further research is necessary.

Monolayer cultures of cell lines and primary cells (two-dimensional (2D) cell culture) are the most commonly used *in vitro* models for investigation of tissues and pathogenesis of diseases, such as cancer, metastases, and infections. However, monolayer cultures are insufficient to capture the complex influence of stromal environment in disease development. Three-dimensional (3D) organotypic models are a promising approach to gain an *in vivo* like understanding of molecular disease development. Various 3D organotypic models are established for a multitude of organs including heart, lung, intestine, pancreas, liver, skin, esophagus, pancreas, prostrate, omentum majus or colon [16–22]. To our knowledge, there is no implemented 3D organoid model of the pleura. The aim of this investigation was to develop a three-dimensional-organotypic model as a basis for the study of pathogenesis and therapeutic regimes for the pleural diseases *in vitro*.

## Materials and methods

### Cell culture

#### Isolation of human pleural mesothelial cells (HPMC)

Specimens of healthy human *pleura parietalis* were obtained from patients undergoing pleurectomy surgery at the University Hospital Leipzig, Germany, Department of Thoracic Surgery. Pleura biopsies were collected from patients free of pathologic conditions. Informed consent was obtained before surgery and the study was approved by the ethics committee of the University of Leipzig (477/20-ek). The tissues were transported in sterile phosphate buffered saline (PBS–Dulbecco’s, Gibco, Germany). Pleura specimens were washed several times in PBS and finally dissected into approximately 2 mm^2^ segments. These were washed in PBS three times to remove any contaminating red blood cells and incubated for 25 min an orbital shaker with PBS and 0.25% trypsin/25 mM EDTA (50:50, v/v) (trypsin/EDTA, Sigma, Germany) at 37°C for 20 min [23]. During establishment process the pleural specimens were subjected to enzymatic disaggregation with higher trypsin concentrations (PBS and 0.25% trypsin/25 mM EDTA (1:2, v/v) or the time of enzyme exposure was increased up to 40 minutes. The cell suspension was centrifuged at 1,500 rpm for 10 min and the pellet was washed twice with 5 ml of RPMI 1640, 20% FBS, 100 U/ml penicillin and 100 μg/ml streptomycin. Mesothelial cells were plated with 5 ml of RPMI 1640, 20% FBS and 100 U/ml penicillin/100 μg/ml streptomycin into one well of 6-well plate. The cells were maintained at 37°C and 5% CO_2_ for five days until confluence was reached. Purification of primary human pleural mesothelial cells (HPMC) was verified by vimentin and cytokeratin positive immunofluorescence staining. Cells at passage 2–5 were used for the experiments.

#### Isolation of human pleural fibroblasts (HPF)

The enzymatic disaggregation of tissue to isolate fibroblasts was modified from *Joerres et al. [24].* Pleural specimens were handled under sterile conditions, washed three times in PBS and pieces approximately 2 mm^2^ in size were incubated in 5 ml trypsin/EDTA/glucose (0.125%/0.01%/0.1 wt/vol in PBS) for 20 min at 37°C an orbital shaker. The solution containing cells in suspension was centrifuged at 1,500 rpm for 10 min. The remaining tissue and trypsin/EDTA/glucose solution were removed, and the pelleted cells were washed twice (1,500 rpm for 10 min) in RPMI 1640 containing 20% FBS to inhibit the trypsin activity. The trypsin/EDTA digestion was repeated once for 20 min after two more digestion steps of 40 min each. The cells obtained during each disaggregation step were resuspended in 5 mL of RPMI 1640, 20% FBS and 100 U/ml penicillin/100 μg/ml streptomycin and transferred to 25 cm^2^ culture flasks. The primary cultures were incubated at 37°C and 5% CO_2_. The medium was exchanged after 4 days for the first time and every third day thereafter. Fibroblast purification was confirmed by prolyl-hydroxylase 1 positive and cytokeratin negative immunofluorescence staining. Cells at passage 2–4 were used for the experiments.

### 2D cell culture

HPMC and HPF cells were isolated from human *pleura parietalis* as described above and cultured in RPMI 1640, 20% FBS and 100 U/ml penicillin/100 μg/ml streptomycin for 7 days with medium changed every 72 hours. Confluent cell cultures were subcultured as follows: the cell cultures were washed with PBS and incubated with trypsin/EDTA solution (0.25% trypsin/25 mM EDTA.) The detachment of cells was observed by light microscopy and the trypsin activity inhibited by adding FBS-supplemented culture medium thereafter. The cells were washed twice in FBS-supplemented culture medium and seeded into new culture flask at one third of the density of the previous culture. For the construction of 3D organotypic co-cultures, HPMC and HPF cells from the same pleura biopsy at passage 2–4 were used. For immunocytochemical and immunofluorescence characterization of HPMC and HPF, cells were grown to confluence in 12-well chamber slides (Nunc, Germany) and fixed in ice-cold methanol/acetone (1:1 vol/vol).

### 3D co-culture model of pleura

#### Gel construction

**Fig 1:**
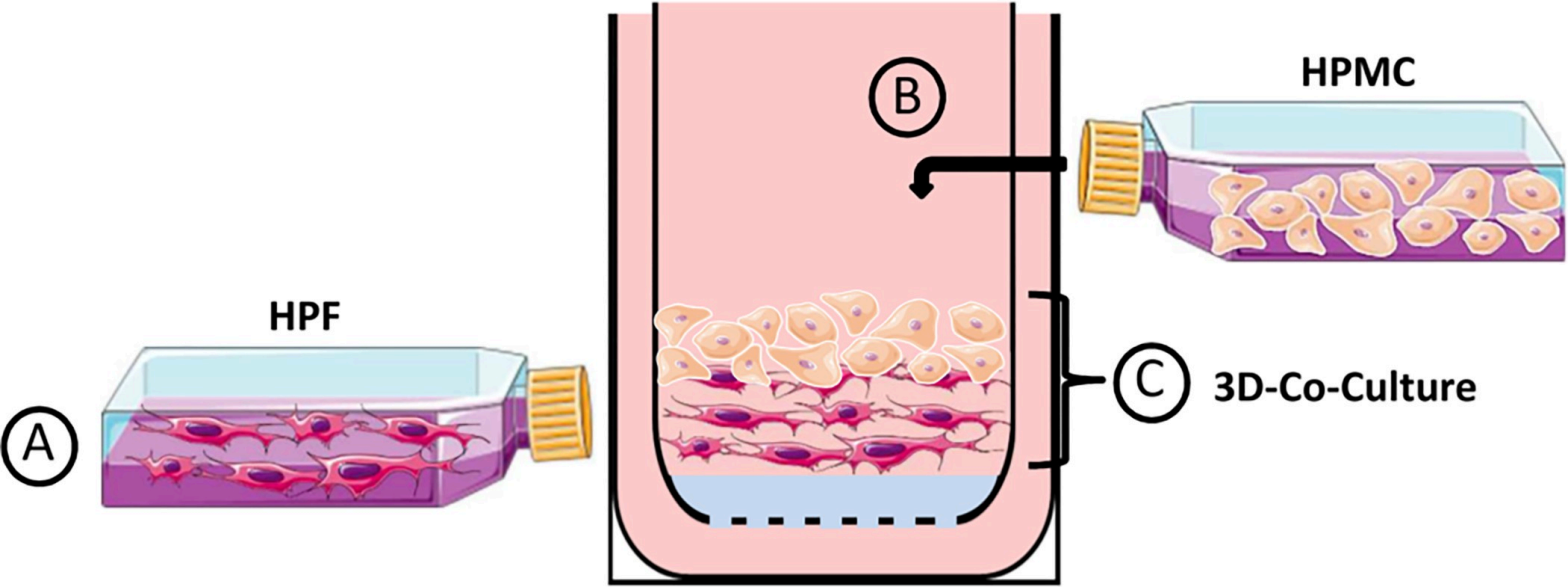
Schematic overview of experimental methods for reconstruction of pleura by using a three-dimensional collagen gel culture. Human pleural fibroblasts (HPF) (a) and human pleural mesothelial cells (HPMC) (b) were isolated and placed into culture flasks. A single cell suspension of fibroblasts obtained from confluent monolayers was passaged and cultivated in a type I collagen gel matrix. After the solidification mesothelial cells were placed on the gel matrix to build up a three-dimensional pleura-like structure (c).

The collagen matrix was prepared by mixing of 1 vol of acid-soluble rat tail collagen HC, Type I (ibidi, Germany) with 8 vol of DMEM media (DMEM, 10% FBS and 100 U/ml penicillin/100 μg/ml streptomycin) and was reconstituted with 1 vol of NaOH buffer (0.1 N NaOH). The 3D collagen gel culture was assembled by plating 5x10^4^ human pleural fibroblast cells inside the gel (Fig 1A), followed by seeding of 3.5x10^5^ human pleural mesothelial cells on the gel to form a co-culture (Fig 1B). HPF from the primary culture were gently mixed and embedded into collagen gel as a single cell suspension. 400 μl of gel with fibroblasts was brought into 12-well inserts with polymer mesh (ThinCert 8 μM pore size, translucent, Greiner Bio-One, Germany) which were placed in into 12-well plates. The collagen solidified at 37°C for 2hr. After solidification collagen gel was coated with 100 μl fibronectin (5 mg/ml) in DMEM without FBS and incubated at 37°C for 1hr. After that, primary mesothelial cells were seeded on the top of the collagen. 1 ml of growth media was added inside the insert, and approximately 2 ml of medium outside the insert and incubated at 37°C until a confluent layer of mesothelial cells formed (Fig 1C).

### Examination of human *pleura parietalis* tissues and 3D co-culture model of pleura

#### Phase-contrast and immunofluorescence

During the culture period morphogenesis and cell growth of primary human pleural cells and cells in 3D co-culture model of pleura were analyzed by phase-contrast and immunofluorescence staining and compared to normal human pleura biopsies. Human *pleura parietalis* was fixed in 4% formalin at 4°C for 24 hr and then paraffin embedded. The paraffin specimens were cut onto Superfrost Plus charged slices (Thermo Fisher Scientific, USA), deparaffinized in xylene and hydrated with alcohol and standard hematoxylin and eosin staining performed.

For immunohistochemical experiments, the peroxidase activity was quenched with 1% H_2_O_2_ in methanol for 10 min. Slides were boiled in 0.01 M sodium citrate pH 6.0 for 10 min to retrieve antigens. The immunofluorescence analysis of the 3D pleura culture and human pleura biopsies were carried out according to the protocol mentioned above. Cells cultured on chamber slides were fixed in acetone and methanol (1:1). All slides were blocked for 1 hr in blocking buffer (130 mM NaCl, 7 mM Na_2_HPO_4_, 3.5 mM NaH2PO4, 7.7 mM NaN3, 0.1% BSA, 0.2% Triton X-100, 0.05% Tween 20 and 10% serum of host of secondary antibody) and incubated overnight at 4°C with primary antibodies against vimentin, PHD1, ZO-1, cytokeratin, Ki-67, β-catenin, VE-Cadherin diluted in blocking buffer. After 3 washes in TBS for 10 min slides were incubated with goat-anti rabbit Alexa Fluor-labeled secondary antibodies diluted in blocking buffer (Table 1). After washing 3 times for 10 min cover ships were mounted with media containing DAPI (Vector Laboratories). Appropriate negative controls for the immunostaining were prepared by omitting the primary antibody. Localization of staining was analyzed by fluorescent microscopy on a Leica Axiovert 100 microscope equipped with Leica digital camera.

**Table 1:** Immunohistochemical reagents.

primary antibodies	sources	dilutions
PHD1	Invitrogen, USA	1:200
VE-Cadherin	Cell Signaling Technology, USA	1:50
vimentin	Cell Signaling Technology, USA	1:100
ZO-1	Invitrogen, USA	1:50
cytokeratin	Abcam, UK	1:400
Ki-67	Cell Signaling Technology, USA	1:50
β-Catenin	Cell Signaling Technology, USA	1:50
**secondary antibodies**		
goat-anti rabbit biotinylated	Abcam, UK	1:200
goat-anti rabbit Alexa Fluor 405	Cell Signaling Technology, USA	1:1000
goat-anti rabbit Alexa Fluor 555	Cell Signaling Technology, USA	1:1000
goat-anti rabbit Alexa Fluor 568	Cell Signaling Technology, USA	1:1000
goat-anti rabbit Alexa Fluor 594	Cell Signaling Technology, USA	1:1000

Table 1 lists of primary and secondary antibodies used.

### Solute permeation test

The permeability of the 3D co-culture model of pleura was determined by measuring changes in the concentration of molecular markers. Fluorescein isothiocyanate-labeled dextran (FITC-labeled dextran) (Sigma-Aldrich, USA) with molecular weight of dextran: 70 kDa and 4 kDa were used as fluorescent markers. 3D pleura culture and fibroblasts in collagen matrix were cultured on 12-well transwell inserts as described above. At specific time points (day 1, day 3 and day 6) after gel construction, the FITC-conjugated dextran and non-FITC conjugated dextran were administered to the upper and lower compartments of the inserts, respectively, maintain isotonic conditions at a final concentration of 50 μg/ml [25]. At 2h after addition of a molecular marker, 100-μl samples were collected from lower compartments of the chamber. Each sample was measured by a fluorescence spectrophotometer (GloMax Discover Microplate Reader, Promega, US) at an excitation wavelength of 490nm and an emission wavelength of 520nm. The FITC-conjugated dextran concentration was calculated using a standard curve. The upper and lower compartments represent the intrapleural and extrapleural spaces, respectively; so, this model represents solute permeation from the intrapleural to extrapleural space.

### Statistical analysis

Data are reported as means ± SD. Differences in mean values were analyzed using t-Test (GraphPad Software, US). Differences were considered significant with *P* < 0.05.

## Results

### Development of a novel 3D co-culture model of pleura

**Fig 2:**
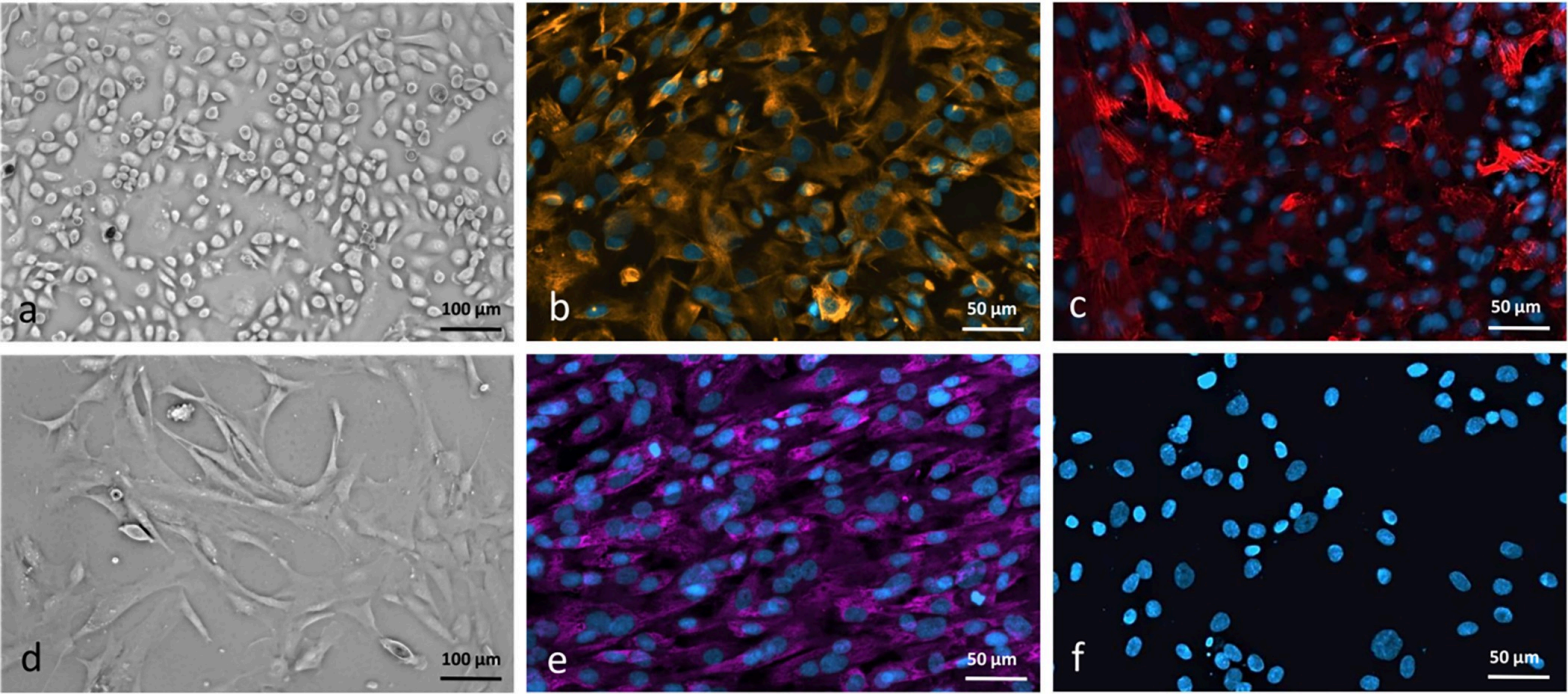
Characterization of human pleural mesothelial cells (HPMC) and human pleural fibroblasts (HPF). Confluent cell cultures of human pleural mesothelial cells (HPMC) (a) and human pleural fibroblasts (HPF) (d). HPMC show polygonal appearance characteristic at confluency, whereas HPF are spindle-shaped cells, growing in parallel, whorl-forming arrays (phase-contrast microscopy; original magnification, x10). Immunofluorescent staining of fixed human mesothelial cells and fibroblasts with (b) monoclonal anti-vimentin and (c) monoclonal anti-cytokeratin for HPMC, x20 and (e) monoclonal anti-PHD1 and (f) monoclonal anti-cytokeratin for HPF, controls where primary antibody has been omitted do not show any unspecific binding, Scale bar = 50 and 100 μm.

After enzymatic disaggregation of human pleura supplies homogeneous mesothelial cells and fibroblasts were assessed by phase-contrast microscopy. A confluent layer of mesothelial cells has a cobblestone appearance (Fig 2A). The mesothelial cells covering normal pleura express the mesenchymal marker vimentin and the epithelial cell marker cytokeratin as well (Fig 2B and 2C) as reported previously [26]. However, increasing the time of enzyme exposure, higher trypsin concentrations and/or incubation temperatures caused a contamination of HPMC culture by fibroblasts, as assessed by cytokeratin positive immunofluorescence staining (for details, please see S1 Fig). Digestion of human pleura supplies for isolation of human pleural fibroblasts was repeated for third cycle to avoid the contamination of mesothelial and other cells, as assessed by phase-contrast microscopy (Fig 2D). A confluent layer of fibroblasts appeared bipolar or spindle-shaped at confluency and showed positive for prolyl hydroxylase 1 and negative for cytokeratin (Fig 2E and 2F). After more than five cell passages, a marked decrease in cell proliferation capacity was noted, and cells began to display morphologic alterations. For this reason, only cells in passage 2–4 cells were used for the construction of the 3D model.

For development of *in vitro* cell culture model that mimic the 3D architecture complexity and phenotypic characteristics of *in vivo* tissue, we optimized several experimental parameters including ratio of the two cell types (HPMC and HPF). We verified H&E stains from 7 *pleura parietalis* biopsies from different patients and found that the ratio of mesothelial cells to fibroblasts is between 6:1 and 8:1. In order to generate a 3D co-culture model of pleura, HPMC and HPF derived from human pleura tissue were plated as described in the Materials and Methods section and graphically illustrated in Fig 1. Therefore, we first mixed HPF with collagen matrix (Fig 1A) and then overlaid this culture with 7 times the number of mesothelial cells (Fig 1B). During the optimization process for the construction of fibroblast layer, the best ratio of collagen to media was 1 vol of collagen to 8 vol of DMEM media. Lower collagen concentration altered the matrix pore size, which altered integrity and functionality. As seen in S2 Fig, 3D co-culture model of pleura with lower concentration of collagen shows no significant change in permeability of the dextran 70 kDa. Assembly of the pleura epithelium atop matrix-embedded fibroblasts suspended on a transwell insert generates a 3D organotypic co-culture that maintains mesothelial cells in close proximity to pleura fibroblasts (Fig 1C).

#### Validation of newly developed 3D co-culture model

In order to enable a direct comparison between our new organotypic model and the existing *in vivo* situation, we examined biopsies of the human *pleura parietalis* in parallel to our 3D co-culture model.

**Fig 3:**
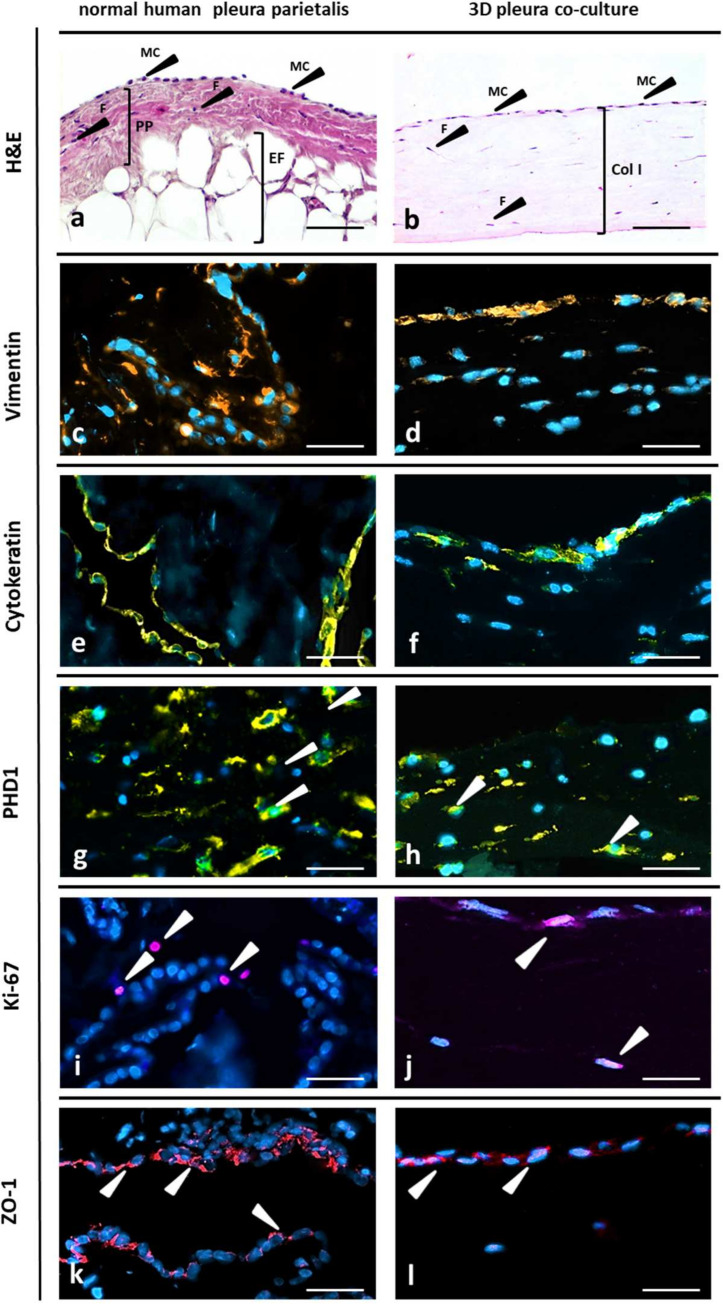
Characterization of normal human pleura and 3D organotypic co-culture of pleura Histology of human *pleura parietalis* (a-k) and of a 3D organotypic model of pleura (b-l). Hematoxylin and eosin staining (H&E) of human *pleura parietalis* (a). PP *pleura parietalis*; MC, mesothelial cells; F, fibroblasts; EF, extrapleural fat. H&E of 3D organotypic co-culture model of pleura. F, pleura fibroblasts; ColI, collagen I matrix; MC, primary human pleura mesothelial cells on top layer (b). Immunofluorescence staining of normal human pleura (c-k) and of 3D organotypic co-culture model (d-l); for vimentin (arrows, orange) (c, d), cytokeratin (yellow) (e, f), PHD1 (arrows, yellow) (g, h), Ki-67 (arrows, purple) (i, j) and ZO-1 (arrows, red) (k, l) with the nuclei counterstained with DAPI (blue). Controls where primary antibody has been omitted do not show any unspecific binding. Scale bar 100 μm.

With hematoxylin and eosin staining, the HPMC were observed to establish a continuous monolayer on the connective tissue with fibroblasts in human pleura *in vivo* (Fig 3A), as did mesothelial cells on the 3D fibroblast matrix in vitro (Fig 3B). The two cell types in the co-culture being in direct contact with each other might better reflect paracrine signal transmission, as has already been proven *in vivo*. In order to enable a direct comparison between our new organotypic model and the existing *in vivo* situation, we examined biopsies of the human *pleura parietalis* in parallel to our 3D co-culture model.

Distribution of markers normally expressed by pleural mesothelium was profiled using immunochemistry and fluorescence staining. Vimentin and Cytokeratin (mesothelial cell marker) (Fig 3C–3F), PHD1 (fibroblast-cell marker) (Fig 3G and 3H), were expressed in the pleura biopsies and showed a similar expression pattern as compared to 3D co-culture model (day 3). Expression of Ki-67 indicates the presence of proliferating cells (Fig 3I and 3J). Tight junctional marker ZO-1 was found localized at contact zones between mesothelial cells (Fig 3K and 3L). Each of these markers was expressed in both the 3D co-culture model and human *pleura parietalis* biopsies.

Histological assessment of the 3D co-culture model compared to human *pleura parietalis* biopsies displayed the presence of both cell types (HPMC and HPF) in the 3D co-culture model which mimic the morphology of the human pleura.

### The permeability of 3D pleura culture was dependent on the mesothelial cell layer

**Fig 4:**
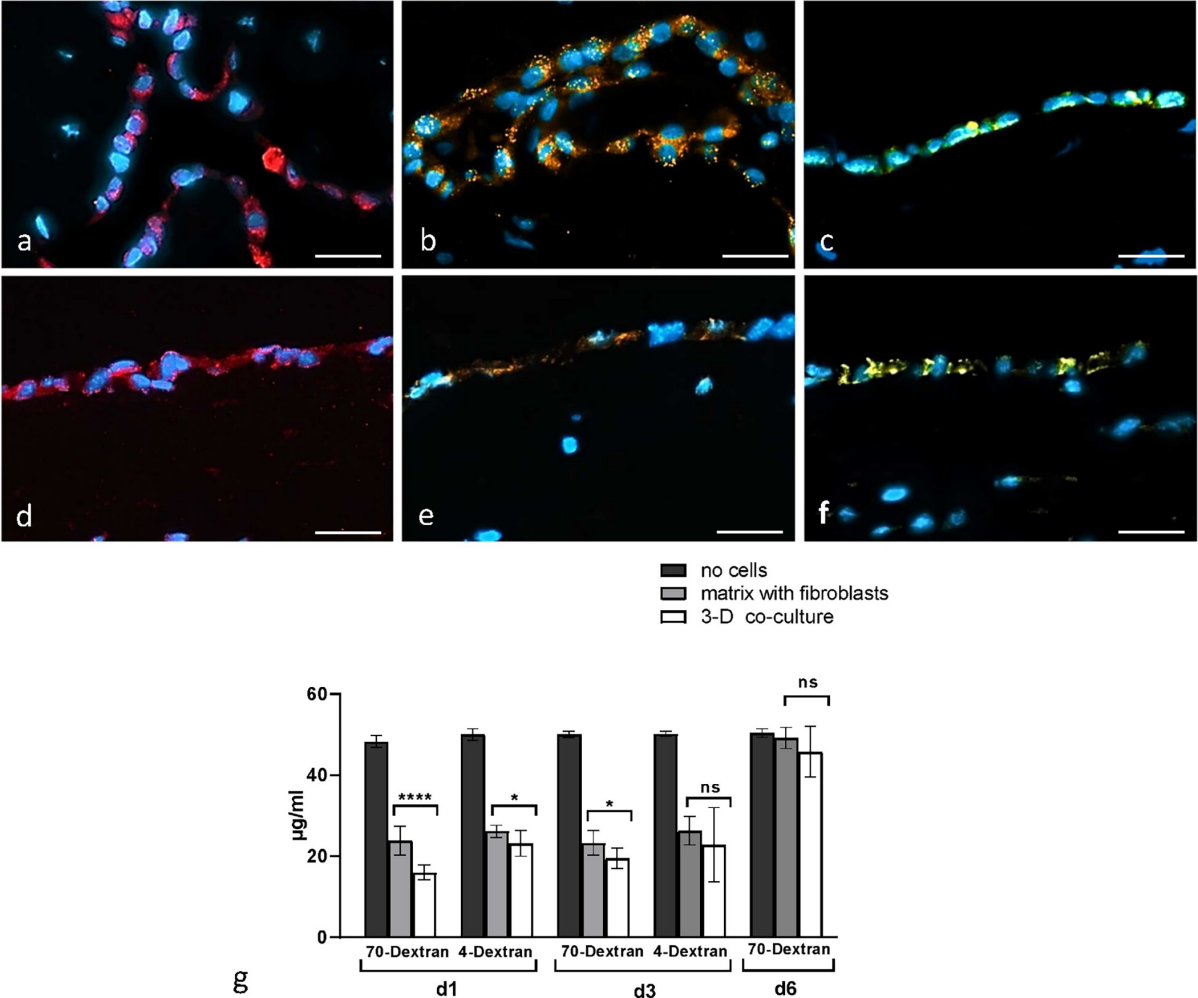
a-g. Expression of junctional proteins and results of permeability assay. Junctional localization of ZO-1, β-Catenin and VE-Cadherin proteins on human normal pleura (a-c) and 3D organotypic co-culture model of pleura (d-f). ZO-1 (red), β-Catenin (orange) and VE-Cadherin (yellow) staining clearly outline cell membranes of HPMC. Nuclei were counterstained with 4, 6-diamidino-2-phenylindole (DAPI) (blue) (a, b, d, e) and hematoxylin (C and f). Human fibroblasts do not show any expression of ZO-1, β-Catenin and VE-Cadherin at cell membranes. Controls where primary antibody has been omitted do not show any unspecific binding. Scale bar = 50μm. Investigation of the permeability of 3D organotypic co-culture model of pleura using FITC-conjugated dextran (g). Vertical axis indicates fluorescein isothiocyanate (FITZ)-conjugated dextran concentration (μg/ml). Horizontal axis shows molecular weight of dextran: 70 kDa and 4 k Da and the age of 3D co-culture model (day 1, day 3 and 6). Fluorescence intensity of FITZ-conjugated dextran leaking from the upper to the lower chambers of transwell membranes was measured in each lower chamber at 2 hours after the addition of a molecular marker. Each bar indicates the mean FITC-conjugated dextran concentration (μg/ml) in the lower chamber. Black, gray and white bars indicate control (transwell, no cells seeded), collagen matrix with fibroblasts and full 3D organotypic model of pleura respectively. Error bars indicate SE. (mean ± SEM; n = 8). *P < 0.05, Student’s t test. This figure is representative of four independent experiments.

Pleura is squamous epithelia of mesothelial origin with different functions of filtration and absorption of pleural fluid. An imbalance in the regulation of these functions can result in an accumulation of fluid between the parietal and the visceral pleura, defined as pleural effusion [27]. In this part, we focus on study the relationship between solute permeability and localization of the tight junction- associated proteins in 3D pleura culture. In order to demonstrate a functional coupling and interaction of the HPM cells with each other, we performed immunohistochemically staining of tight and adhesion junctional proteins: ZO-1, β-Catenin and VE-Cadherin. Fig 4 shows the expression pattern of junctional proteins in human *pleura parietalis* biopsies (Fig 4A–4C) and 3D co-culture model (Fig 4D–4F), tight and adhesion junctional proteins were aligned along the cell membrane between neighboring HPMC. As expected, staining for ZO-1, β-Catenin and VE-Cadherin was negative along the membrane of HPF confirming the absence of functional tight and adhesion junction proteins in fibroblasts (Fig 4A–4F). Morphological analysis of tight and adhesion junctional proteins demonstrated that HPMC can polarize and form well-developed intercellular junctions in our 3D organotypic co-culture model of pleura (Fig 4A–4F). Immunofluorescence labeling/microscopy was complemented by permeability assay (Fig 4G). We investigated the permeability of 3D organotypic co-culture model of pleura using FITC-conjugated dextran. As shown in Fig 4G, solute permeability of the 3D pleura at the day 1 was significantly lower compared to collagen matrix with fibroblasts, indicating that 3D organotypic co-culture model of pleura is more resistant to permeation by middle (70 kDa) and small (4 kDa) molecular weight solutes than fibroblasts in collagen matrix. To search whether the permeability of 3D organotypic co-culture model of pleura changes with the age of the model, permeability assay was performed at the day 3 and day 6 after full 3D pleura model construction. At the day 3, the permeability of 3D organotypic co-culture model of pleura was found to be higher for 70 kDa dextran and no significant difference could be detected in the permeability for small dextran (4 kDa) between both collagen matrix with fibroblasts and full 3D pleura culture model. As distinctly seen in Fig 4G, control (the permeability of polymer mesh without cells) shows no change in the concentrations of any of the dextran (4 kDa, 70 kDa) in the bottom of the opposing well.

We found that solute permeability of the 3D pleura at day 1 was significantly lower compared to days 3 and 6. However, no significant difference could be detected in the permeability for small dextran (4kDa) between both collagen matrix with fibroblasts and full 3D pleura culture model at the day 3. As distinctly seen in Fig 4G, day 6 shows no change in the concentrations of dextran in the bottom of the opposing well between control (polymer mesh without cells) and both collagen matrix with fibroblasts and full 3D pleura culture model.

## Discussion

Pleura represents an epithelium of mesothelial origin which is lining lungs and chest cavity. Monolayer of mesothelial cells delimit parietal as well as visceral pleura from the underlying structures and has multiple key functions regarding transport and barrier properties. Mesothelial cells have a decisive role in inflammation of pleura through releasing several cytokines [28]. Pleural integrity has a central role in barrier maintenance. There is significant evidence that mesenchymal interactions are key drivers for keeping small amounts of pleural liquid which volume and composition is tightly regulated [29]. An imbalance of specific barrier properties of the mesothelial cell layers can result in an accumulation of fluid between the parietal and the visceral pleura, a condition defined as pleural effusion. The ability to understand the complex physiology and behavior of mesothelial cells has been limited by the difficulty of isolation and culturing of human pleural mesothelial cells (HPMC) until today. Therefore, the development of a 3D organotypic co-culture model that preserves direct contact between HPMCs and human pleural fibroblasts (HPF) is a critical step toward developing *in vitro* models of pleura. The herein presented model allows for the understanding of essential drivers of epithelial–mesenchymal cell interactions by allowing access to each cell type within a co-culture for up to 3 days. The newly developed 3D co-culture model of pleura exhibited *in vivo*-like structural and phenotypic characteristics, including the three-dimensional architecture, apical-basolateral polarity, well-formed tight- and adhesion junctions. Our data have demonstrated that our 3D co-culture model of pleura shares key characteristics with the origin organ (pleura) from which it was derived, by expression of the phenotypic markers normally expressed by pleural mesothelium as described in previous studies [27, 30, 31]. The functional properties of 3D co-culture model of pleura were determined by the expression structures which link the epithelial cells together, e.g. adhesion junction protein VE-cadherin, ZO-1 a linker protein and β-catenin one of components of cell–cell adhesion machinery [32–36]. We demonstrated that HPMC successfully form tight and adhesion junctions which is in concordance with previously published works using HPMC monolayer as a model to assess the functional integrity of the peritoneum [37] and pathogenesis of pneumothorax [38] underlying the importance of our established *in vitro* system. We assessed the permeability of our system and detected decrease in permeability of 3D organotypic co-culture model of pleura compared to collagen matrix with fibroblasts and polymer mesh without cells up to age of 3 days after complete construction of 3D co-culture model. However day 6 shows no changes in the concentrations of the dextran in the bottom of the opposing well. This result demonstrates changes of the permeability of 3D organotypic co-culture model of pleura with increasing age of the model. These data strongly support that our 3D organotypic co-culture model of pleura does not have any function as a barrier layer 6 days after model construction. However, the first changes in permeability can be seen as early as three days after construction for small (4kDa) molecular weight solutes. This suggests that 3D organotypic co-culture model of pleura, is less permeable to middle molecular weight solutes until day 3 after construction and however loses its integrity and functionality with increasing age. For this reason, in all subsequent studies, our 3D organotypic co-culture model of pleura was used until day 3 after full 3D construction.

These results demonstrate limitations of our model: the permeability of the model decreases and looses integrity and functionality with increasing age. However, the proliferation of fibroblasts’ growth in the 3D matrix might be a reason for this. Proliferating fibroblasts have tensile forces that effect the collagen-matrix layer. Under the influence of the fibroblasts, the matrix contracts and separates from the plastic of the transwell insert. During the optimization process for the construction of fibroblast layer, changing the stiffness and composition of the collagen-matrix may solve this obstacle and increase the longevity of the co-culture. Collagen is a major component of extracellular matrix (ECM) of connective tissue and provides a highly biocompatible environment for cells. Lu et al. previously characterized the structural and mechanical properties of pleura tissue in detail: pleura has an abundance of elastin and collagen that can serve as a potential biomaterial for 3D tissue reconstruction. *In vivo*, HPMC and HPF grow in a three-dimensional environment composed of collagen. The use of collagen scaffold is the best way to reconstitute the structural and mechanical characteristics of human pleura tissue [39, 40]. By *in vitro* cultivation, a 3D environment simulates in vivo situation better than 2D cell culture [41]. Collagen allows cell attachment through integrin receptors, which control cell survival, growth, and differentiation [42]. This makes collagen suitable as biomaterial for our 3D co-culture of pleura for modeling biological ECM.

Our experimental method suggests the role of mesothelial cells in transpleural transport and their association in the formation of cell-cell adhesion of the mesothelial monolayer in 3D organotypic co-culture model of pleura. Immunofluorescence analysis of our 3D organotypic co-culture model of pleura showed equal distributions of ZO-1, β-Catenin and VE-Cadherin, which are associated with pleural permeability, along the membrane at cell-cell contacts. The role of the mesothelial cells in pleural fluid removal and vesicular transport of protein under physiological conditions is supported through a series of realizations and considerations [43–45]. In contrast to HPMC monolayer human pleura fibroblasts do not express ZO-1, β-Catenin or VE-Cadherin [46, 47]. Our findings to permeability of 3D organotypic co-culture model of pleura correlated very well with data of Bodega et al. in the study of macromolecule transfer through mesothelium and connective tissue [48]. The 3D co-culture model of human pleura that was engineered, validated, and tested in this study represents a new tool to investigate mechanisms underlying diseases with both non-infectious and infectious etiologies and tumorigenesis. This model offers a wide range of applications covers basic science for gaining further knowledge on the development and characteristics of pleural pathologies and the evaluation of approaches, such as antiseptic solutions, antibiotics, or tumor therapy. 3D co-culture models offer advantages for drug screening to study morphogenesis, lineage commitment, and tissue regeneration. An organoid model is fast in implementation and moderately inexpensive with good correlation within *in vivo* clinical studies and a reproducibility of results. They may accelerate endeavors in reducing animal testing in sciences. Significant differences in histology and immunology of rodents and humans reduce explanatory power of rodent models. Besides, non-standardized study protocols impair reproducibility of study results [49]. 3D co-culture models proved as a reliable alternative for animal models for a large number of organs, including intestine, liver, pancreas, bones, heart, skin and even lung [17, 18, 22, 50–53]. Our 3D organotypic co-culture model of pleura can be a useful tool to understand mesothelial infections and evaluate and enhance therapeutic strategies, such as antibiotics or antiseptic solutions. In an approach to model infectious disease, our group is planning experiments to mimic *Staphylococcus aureus* infection and biofilm development in pleural cavities in this model. Our model will be used for investigations of the impact of different environmental parameters on colonization as well as co-localization patterns of pathogens with different host cell types. We believe that our 3D organotypic co-culture model of pleura can not only provide valid data on the biofilm pathology and host-pathogen interactions but can also potentially be used to demonstrate the effect of antibacterial agents to investigate their impact on pleural bacterial load. In summary, the presented 3D co-culture model of pleura functions as a robust assay for pleural research serving as a precise reproduction of the *in vivo* morphology and microenvironment. To our knowledge, it is the first reproducible 3D assay of the pleura which constitutes it as a valuable and novel tool for development of preventive and therapeutic enhancement of various pleural disease.

## Supporting information

S1 Fig Determination of human pleural mesothelial cells (HPMC) purity by cytokeratin staining. Suitable ratio of trypsin concentration and incubation time was determined by various protocols. Higher trypsin concentration led to increased contamination of cells without cytokeratin expression (a). Longer time of trypsin exposure similarly increased contamination of cells without cytokeratin expression (b). Immunofluorescent staining of HPMC according to standard digestion protocol with monoclonal anti-cytokeratin showed acceptable amount of contamination (c). Anti-cytokeratin (arrows, red), DAPI nucelar staining (blue). Scale bar = 50 μm.
(TIF)

S2 Fig Results of permeability assay on optimization process for the construction of fibroblast layer of 3D co-culture model of pleura. *Vertical axis*: fluorescein isothiocyanate (FITZ)-conjugated dextran concentration (μg/ml) *Horizontal axis*: concentration of collagen in fibroblast layer (1-vol and 0.5-vol). Fluorescence intensity of FITZ-conjugated dextran leaking from the upper to the lower chambers of transwell membranes was measured in each lower chamber at 2 hours after the addition of a molecular marker. Each bar indicates the mean FITC-conjugated dextran concentration (μg/ml) in the lower chamber. Black, gray and white bars indicate control (transwell, no cells seeded), collagen matrix with fibroblasts and full 3D organotypic model of pleura respectively. Error bars indicate SE. (mean ± SEM; n = 8). *P < 0.05, Student’s t test. This figure is representative of four independent experiments.
(TIF)
