# Supplementary figures and images for: Novel 3D organotypic co-culture model of pleura
Source: PLoS One. 2022 Dec 1;17(12):e0276978. doi: 10.1371/journal.pone.0276978 (PMC9714887; doi:10.1371/journal.pone.0276978)

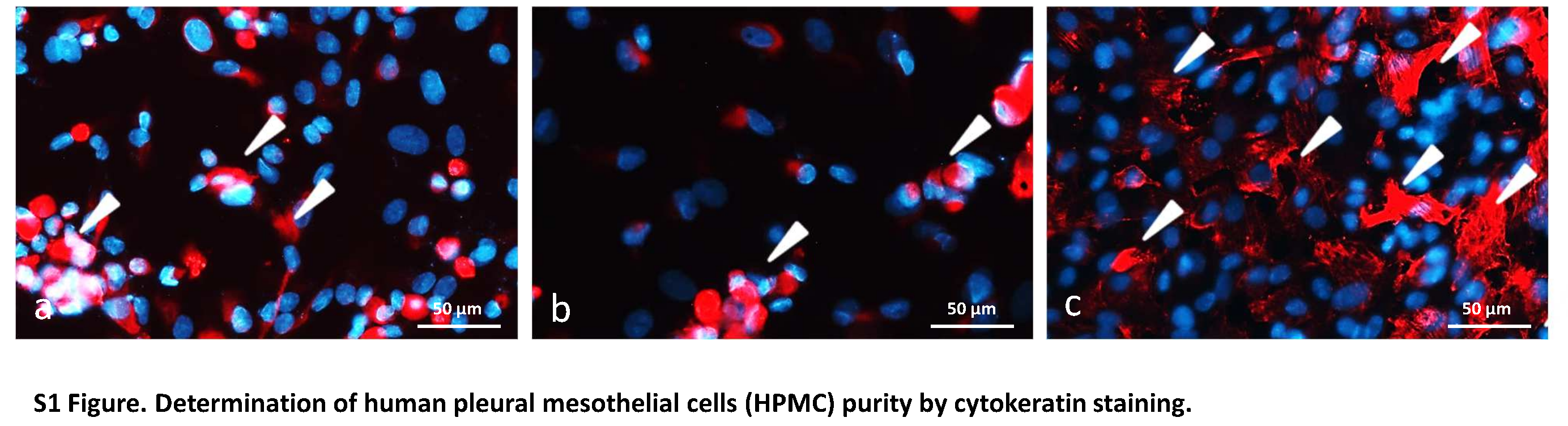

Supplement: S1 Fig — Suitable ratio of trypsin concentration and incubation time was determined by various protocols. Higher trypsin concentration led to increased contamination of cells without cytokeratin expression (a). Longer time of trypsin exposure similarly increased contamination of cells without cytokeratin expression (b). Immunofluorescent staining of HPMC according to standard digestion protocol with monoclonal anti-cytokeratin showed acceptable amount of contamination (c). Anti-cytokeratin (arrows, red), DAPI nucelar staining (blue). Scale bar = 50 μm. (TIF) [file pone.0276978.s001.tif]

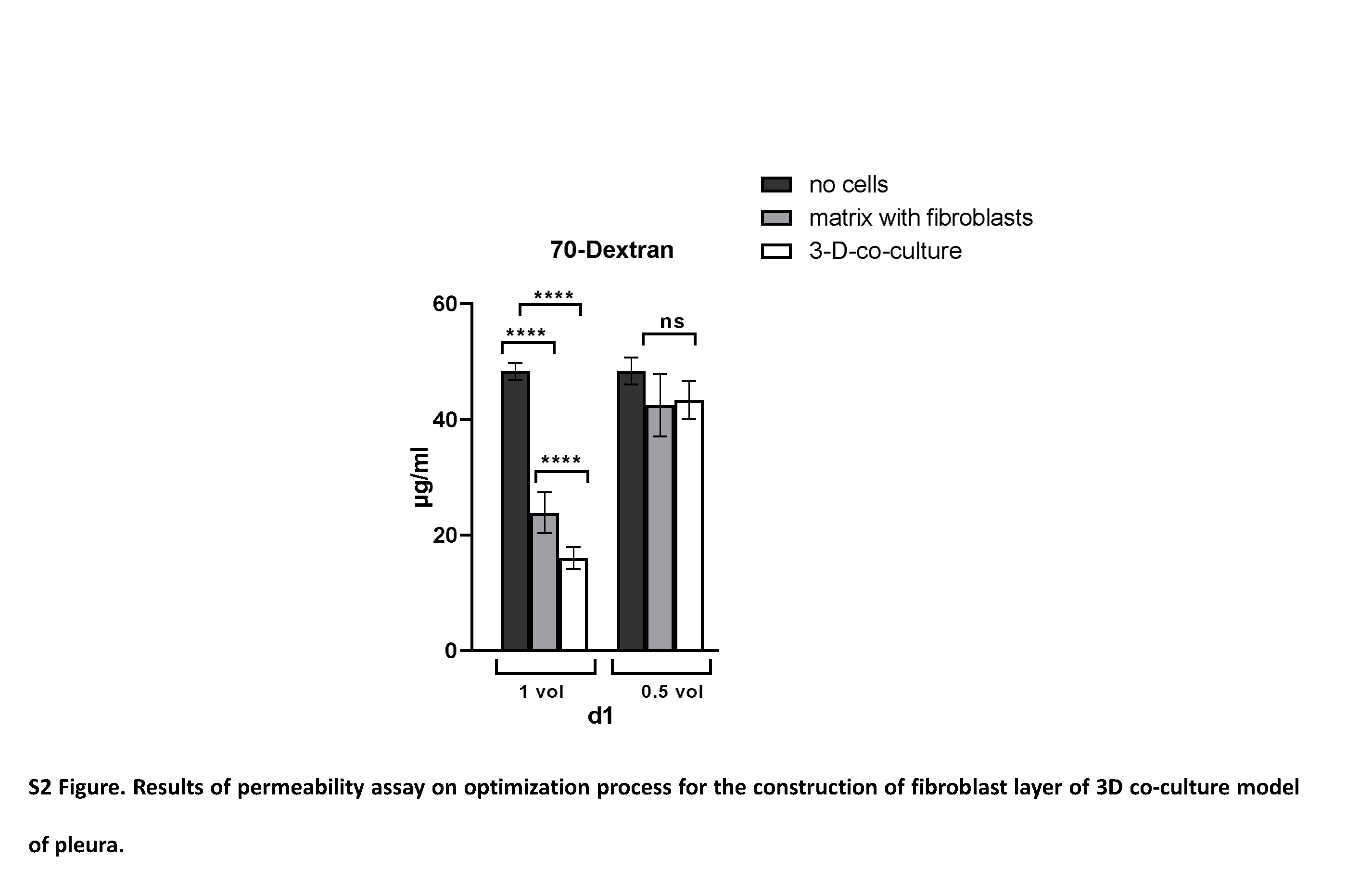

Supplement: S2 Fig — Vertical axis: fluorescein isothiocyanate (FITZ)-conjugated dextran concentration (μg/ml) Horizontal axis: concentration of collagen in fibroblast layer (1-vol and 0.5-vol). Fluorescence intensity of FITZ-conjugated dextran leaking from the upper to the lower chambers of transwell membranes was measured in each lower chamber at 2 hours after the addition of a molecular marker. Each bar indicates the mean FITC-conjugated dextran concentration (μg/ml) in the lower chamber. Black, gray and white bars indicate control (transwell, no cells seeded), collagen matrix with fibroblasts and full 3D organotypic model of pleura respectively. Error bars indicate SE. (mean ± SEM; n = 8). *P < 0.05, Student’s t test. This figure is representative of four independent experiments. (TIF) [file pone.0276978.s002.tif]
